# Associations Between State Policies Facilitating Telehealth and Buprenorphine Episode Initiation and Duration Early in the COVID Pandemic: State Telehealth Policies and Buprenorphine

**DOI:** 10.1007/s11606-024-09188-6

**Published:** 2024-11-14

**Authors:** Bradley D. Stein, Brendan K. Saloner, Flora Sheng, Mark Sorbero, Andrew W. Dick, Adam J. Gordon

**Affiliations:** 1 RAND Corporation, Pittsburgh, USA; 2 Johns Hopkins Bloomberg School of Public Health, Baltimore, MD USA; 3 RAND Corporation, Arlington, VA USA; 4 RAND Corporation, Boston, MA USA; 5 Program for Addiction Research, Clinical Care, Knowledge and Advocacy, Division of Epidemiology, Department of Internal Medicine, University of Utah School of Medicine, Salt Lake City, USA; 6 Informatics, Decision-Enhancement, and Analytic Sciences Center, VA Salt Lake City Health Care System, Salt Lake City, USA

**Keywords:** Buprenorphine, Opioid use disorder, Telehealth, State policies

## Abstract

**Importance:**

State policies facilitating telehealth implemented early in COVID may support buprenorphine treatment of opioid use disorder. However, little empirical information is available about those policies’ effects.

**Objective:**

Examine association between state policies that may facilitate telehealth use and buprenorphine treatment.

**Design, Setting, Participants:**

Retrospective cohort study using 2019–2020 national pharmacy data on dispensed buprenorphine prescriptions.

**Exposures:**

State policies implemented after March 3, 2020, public health emergency declaration requiring private insurers’ telehealth reimbursement to be commensurate with in-person service reimbursement, authorizing Medicaid reimbursement for audio-only telehealth, allowing physicians to provide cross-state telehealth services, and allowing psychologists to provide cross-state telehealth services.

**Main Outcomes and Measures:**

(a) Duration of treatment episodes started between March 1 and March 13 in both 2019 and 2020, and (b) daily numbers of new buprenorphine treatment episodes from March 13 through December 31 in each year.

**Key Results:**

We found little change in the number of new buprenorphine treatment episodes started in 2020 compared to 2019 and an increase in treatment duration of 10.3 days (95%CI 8.3 to 12.2 days) for episodes started in March 2020 before the public health emergency declaration compared to the comparable 2019 period. States implementing a telehealth parity policy in 2020 had 7.3% (95%CI − 13.3% to − 0.4%) fewer new buprenorphine treatment episodes. States joining the psychologist interstate compact in 2020 after the public health emergency declaration had treatment episodes 7.97 days longer (95%CI 0.78 to 15.16) than other states. None of the other policies examined was associated with changes in new treatment episodes or treatment duration.

**Conclusions and Relevance:**

Policies undertaken during the pandemic we examined were associated with few significant changes in buprenorphine treatment initiation and duration. Findings suggest realizing the benefits of telehealth and other policy changes for buprenorphine may require more extensive implementation and infrastructure support.

## INTRODUCTION

The COVID-19 pandemic disrupted healthcare delivery across the USA.^1–4^ It also stimulated higher rates of drug use and misuse,^5–7^ emergency medical system calls, and emergency department visits for opioid overdose.^8–11^ These contributed to historically high rates of fatal overdoses, with estimates of over 107,000 fatal overdoses annually in 2022, little changed from the estimated number of fatal overdoses 2021.^12^

To facilitate access to treatment during a time of social distancing, federal rules allowed state policymakers to extend existing policies and enact new ones to facilitate telehealth treatment for behavioral health disorders. These included multiple policies regarding reimbursement for telehealth services, including both providing telehealth behavioral health services and parity in payment (i.e., requiring that virtual and in-person visits be reimbursed at the same rate).^1,13–17^ Many states also participated in interstate compacts enabling clinicians beyond just those licensed in the state to provide services to state residents via telehealth, including the Interstate Medical Licensure Compact (IMLC) for physicians and the Psychology Interjurisdictional Compact (PSYPACT) for psychologists. Policies facilitating telehealth treatment were synergistic with policies implemented during COVID to increase access to opioid use disorder (OUD) treatment via telehealth, such as policy changes allowing clinicians to initiate buprenorphine treatment without an in-person visit.^17,18^

While delivery of physical health care and treatment for alcohol use disorder declined in the early month of the pandemic compared to prior years,^2,19^ buprenorphine treatment for OUD remained relatively unchanged during the pandemic,^20–26^ explained in part by a decrease in the number of individuals initiating buprenorphine treatment,^21,22^ offset by fewer individuals receiving buprenorphine treatment ending their treatment episode.^27^ It is unclear how policies designed to facilitate telehealth may have contributed to patterns of buprenorphine treatment for OUD at the population level. Studies have explored how telehealth might support treatment and buprenorphine prescribing for individuals with OUD,^28–30^ examining telehealth use by buprenorphine-prescribing clinicians,^30^ clinician perceptions of telehealth’s benefits and challenges,^31–33^ growth in telehealth services provided in substance use disorder treatment facilities,^34^ effects of telehealth on retaining individuals in treatment,^35–37^ and use of buprenorphine by Medicare beneficiaries.^38^ Many of these studies identified potential benefits of telehealth; other studies suggest that uptake of telehealth among many buprenorphine prescribers may have been modest, with limited effects on care delivery.^39^

However, few studies have examined how policies requiring telehealth payment parity or allowing audio-only telehealth affected dispensing of buprenorphine. Nor have studies examined how other policies that facilitated use of telehealth may have influenced dispensing of buprenorphine. Buprenorphine, the most widely used medication to treat opioid use disorder, improves quality of life and decreases fatal overdose rates and all-cause mortality for individuals with opioid use disorder.^40–43^ Greater access to waivered buprenorphine prescribers and receipt of concurrent non-pharmacologic treatment have both been associated with increased use of buprenorphine,^44^ and the interstate compact allowing physicians to practice across state lines could allow individuals to receive buprenorphine prescriptions from physicians licensed in other states. Similarly, individuals receiving buprenorphine could receive therapy and counseling from psychologists licensed in other states in states participating in the psychologist interstate compact.

To address this gap in the literature, we used national retail pharmacy claims data and information about state policies regarding telehealth payment parity, audio-only telehealth services, interstate compacts allowing physicians and psychologists to practice across state lines, to examine the association between state policies and buprenorphine treatment during the pandemic, and to compare those patterns to pre-pandemic patterns. We examined how many buprenorphine treatment episodes began in the months following the declaration of the public health emergency in 2020 and how those numbers differed from the analogous period in 2019 in states that did and did not have policies, as well as examining the duration of treatment episodes initiated immediately before the declaration of the public health emergency. We hypothesized that these state policies would be associated with longer buprenorphine treatment episodes and higher rates of new buprenorphine treatment episodes.

## METHODS

**Fig. 1:**
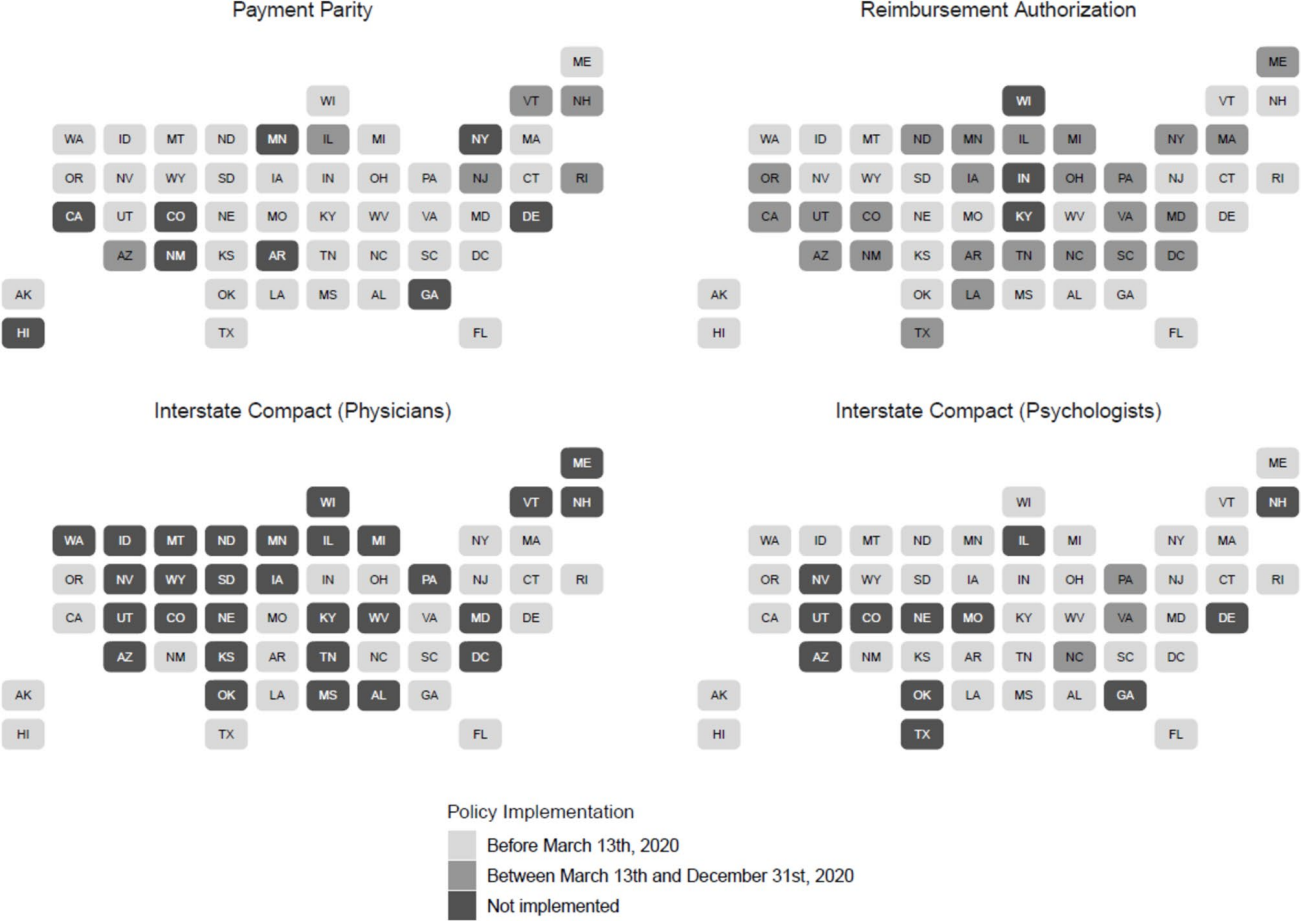
Implementation of policies across states

We used 2019 and 2020 de-identified pharmacy claims from the IQVIA Real World Data – Longitudinal Prescriptions^45^ to identify buprenorphine treatment episodes. These data capture an estimated 92% of all prescriptions dispensed at retail pharmacies in all 50 states and the District of Columbia. We focused on four state policies associated with an increase in behavioral health treatment facilities offering telehealth services,^46^ including (1) policies requiring private insurers’ reimbursement for telehealth to be commensurate with those for in-person services hereafter “telehealth parity,” which researchers had previously identified and cross-referenced from four sources: Manatt,^47^ the American Psychological Association,^48^ the Center for Connected Health Policy,^49,50^ and individual state legislative websites;^46^ (2) the Interstate Medical Licensure Compact (IMLC), which allows physicians to provide cross-state telehealth services and thereby might increase the number of clinicians prescribing buprenorphine to treat OUD in a state;^51^ (3) the Psychology Interjurisdictional Compact (PSYPACT), which allows psychologists to provide cross-state telehealth services,^52^ and might increase the availability of therapy and counseling services available to patients receiving buprenorphine; and (4) Medicaid reimbursement for audio-only telehealth services. Figure 1 depicts for each of the four policies states for which a policy was implemented prior to March 13, 2020; states that implemented the policy during 2020 after the declaration of the public health emergency on March 13; and states in which the policy had not been implemented before the end of 2020.

We identified individuals starting a new buprenorphine treatment episode in March prior to the declaration of the COVID public health emergency (March 1 to March 13, 2020, as well as individuals starting a new buprenorphine treatment episode from March 14 through December 31 after the declaration of the public health emergency on March 13). A new treatment episode was defined as starting the date an individual was dispensed a buprenorphine formulation with an FDA indication for OUD treatment following at least a 60-day period in which the individual received no dispensed buprenorphine, and any days’ supply from a prior prescription was exhausted, consistent with prior research.^27,53,54^ An episode ended when the days’ supply of buprenorphine from dispensed prescriptions was exhausted, followed by at least 60 days with no new dispensed buprenorphine prescription, or as of the last day in the year the episode started, whichever was later.^27,53,54^ State was assigned using the location of the prescriber of the buprenorphine episode’s first dispensed prescription. The study was approved with a waiver of consent by the RAND IRB; STROBE Guidelines for reporting observational studies were followed.

### Analysis

To better understand the association between the change of status of state policies during COVID-19 and initiating new buprenorphine treatment episodes, we compared new treatment episodes from March 14 to December 31, 2020, to the same period in 2019. For each of the four policies we examined, we calculated the relative mean difference in new buprenorphine treatment episodes in states that (a) never had the policy, (b) implemented the policy prior to the declaration of the public health emergency, and (c) did not have the policy implemented prior to the public health emergency but implemented it in 2020 after the public health emergency.

To examine the association of state policies with buprenorphine episode treatment duration, we focused on the cohort starting treatment in March prior to the declaration of the public health emergency in 2020 (March 1–13, 2020). For each of the four policies we examined, we first compared the mean differences in buprenorphine treatment episode duration in days for episodes starting between March 1, 2020, and March 13, 2020, and between March 1, 2019, and March 13, 2019, in states that (a) never had the policy, (b) had the policy both prior to and after the declaration of the public health emergency, and (c) implemented the policy in 2020 after the public health emergency. We then estimated ordinary least square regressions for the treatment duration in days for episodes starting for these 2-week periods during 2019 and 2020, controlling for the change of status of each policy and states fixed effect, with robust error terms clustered at the state level. State participation in the IMLC was excluded from the regression analysis as there was no state IMLC policy change during 2020, as all states participating in the IMLC had started participating in 2019 or before.

## RESULTS

### New Treatment Episodes

We identified 580,931 buprenorphine treatment episodes started from March 14 to December 31, 2019. Consistent with Fig. 1, for each of the policies, there were more episodes in states that had implemented the policy before March 13, 2020, than episodes in states who had not implemented the policy by that time. (see Appendix, Table 4 for details) There were 551,752 buprenorphine treatment episodes started from March 14 to December 31, 2020, a non-statistically significant decrease of 2.1% episodes (*n* = 29,179) (95%CI − 5.5 to 1.4%) (Table 1). States that implemented a telehealth parity policy after March 13, 2020, had 7.3% (95%CI − 13.3 to − 0.4%) fewer new buprenorphine treatment episodes from March 14 to December 31, 2020, compared to March 14 to December 31, 2019; neither having a telehealth parity policy in place prior to March 2020, nor not implementing one prior to December 31, 2020, was associated with a significant change in the number of new buprenorphine treatment episodes from March 14 to December 31, 2020, compared to March 14 to December 31, 2019. Nor were any of the other policies examined (Medicaid audio-only telehealth authorization, physician interstate compact, psychologist interstate compact) associated with changes in the number of new buprenorphine treatment episodes started from March 14 to December 31, 2020, compared to the same period in 2019.

**Table 1:** Difference in Number of New Buprenorphine Treatment Episodes Started Between March 14 and December 31, 2019, and March 14 and December 31, 2020

Policy	Overall		Never implemented		Implemented policy during 2020		Implemented prior to March 13 2020	
	Mean	95% CI	Mean	95% CI	Mean	95% CI	Mean	95% CI
Telehealth parity law	− 2.1%	(− 5.5%, 1.4%)	− 1.5%	− 5.9%, 3.0%	− 6.9%	− 13.3%, − 0.4%	− 1.2%	(− 11.0%, 8.6%)
Medicaid reimbursement for audio-only telehealth services	− 2.1%	(− 5.5%, 1.4%)	− 1.7%	− 7.0%, 3.7%	− 2.7%	(− 8.6%, 3.2%)	0.0%	(− 9.2%, 9.1%)
Physician compact (IMLC)	− 2.1%	(− 5.5%, 1.4%)	− 4.5%	− 9.2%, 0.1%	n/a	n/a	− 0.2%	(− 5.3%, 4.9%)
Psychologist compact (PSYPACT)	− 2.1%	(− 5.5%, 1.4%)	− 1.1%	− 5.7%, 3.5%	− 9.0%	(− 19.7%, 1.7%)	− 3.2%	(− 8.8%, 2.5%)

### Treatment Duration

We identified 28,722 buprenorphine treatment episodes started during March 1–13, 2019, with a mean duration of 139.3 days (SD = 120.7) and 25.8% of episodes censored at the end of the year. The mean duration of treatment episodes started during March 1–13, 2019, was comparable in states that had implemented each policy before March 13, 2020, with states that had not implemented each policy by that time (see Appendix, Table 4 for details). There were 31,560 buprenorphine treatment episodes started during March 1–13, 2020, with a mean duration of 149.5 days (SD = 122.0) with 29.2% of episodes censored at the end of the year, an increase of 10.3 days (95%CI 8.3 to 12.2 days) (Table 2). Descriptively, we did not observe clear patterns for increased duration of buprenorphine episodes starting in those weeks in 2020 in relation to state policy status for the policies we examined (Table 2).

**Table 2:** Mean Difference in Duration of Buprenorphine Treatment Episodes Started Between March 1 and March 13, 2020, and March 1 and March 13, 2019

	Overall		Never implemented		Implemented policy during 2020		Implemented prior to March 13 2020	
	Mean	(95% CI)	Mean	(95% CI)	Mean	(95% CI)	Mean	(95% CI)
Telehealth parity law	10.3	(8.3, 12.2)	11.4	(9.1, 13.6)	9.7	(2.7, 16.7)	6.3	(1.9, 10.8)
Medicaid reimbursement for audio-only telehealth services	10.3	(8.3, 12.2)	5.7	(2.0, 9.4)	12.3	(9.9, 14.8)	8.9	(2.2, 15.7)
Physician compact (IMLC)	10.3	(8.3, 12.2)	8.7	(6.1, 11.3)	n/a	n/a	12.2	(9.3, 15.1)
Psychologist compact (PSYPACT)	10.3	(8.3, 12.2)	10.1	(7.7, 12.4)	18.3	(13.1, 23.6)	4.8	(0.0, 9.6)

Confidence intervals were obtained using pooled method to account for equal variances between cohorts

However, a multivariable regression that included the three policies that states implemented in 2020 subsequent to the declaration of the public health emergency with state fixed effects examining the relationship between year, change of state policy status, and duration of the buprenorphine treatment episodes started during March 1–13 found that states joining the psychologist interstate compact in 2020 after the declaration of the public health emergency was associated with treatment episodes 7.97 days longer (95%CI 0.78 to 15.16 days) compared to states without a status change in being part of the compact (Table 3). We did not find evidence of significant associations between increased treatment duration and change in policy status for telehealth parity laws or Medicaid audio-only telehealth authorization, and no states changed their policy with respect to the interstate physician compact in 2020 after the declaration of the public health emergency.

**Table 3:** Multivariable Regression Results Examining Duration of Buprenorphine Treatment Episodes Initiated Between March 1 and March 13, 2020, and State Policies

	Estimates	95% CI	*p*
Psychologist interstate compact			
Joined after PHE	7.97	(0.78, 15.16)	0.0304
No change	Ref		
Telehealth parity law			
Changed after PHE	− 0.37	(− 7.35, 6.62)	0.916
No change	Ref		
Medicaid audio-only telehealth authorization			
Changed after PHE	3.42	(− 3.89, 10.72)	0.352
No change	Ref		
Year			
2020	7.08	(1.87, 12.30)	0.009
2019	Ref		

Standard errors are clustered at the state level

## DISCUSSION

During the early days of the COVID pandemic, there were substantial concerns about access to medication treatment for opioid use disorder and worries about the consequences of decreased access to healthcare services in general.^55,56^ Comparing the first 9 months of the public health emergency to the comparable period in 2019, we found a very slight decrease in new buprenorphine treatment episodes, and a modest increase in treatment duration for individuals starting treatment right before the declaration of the public health emergency. Our findings in multi-payer populations extend prior research in commercial and Medicaid populations^20,23,57^ suggesting that buprenorphine treatment did not meaningfully change during the pandemic in a manner analogous to decreases in other areas of healthcare.^2^ Similarly, our finding that individuals already being treated with buprenorphine remained in treatment significantly longer in 2020 compared to 2019 is consistent with other studies.^38,58^

To facilitate telehealth treatment during the pandemic, multiple states implemented a range of policies, including those approving reimbursement for audio-only telehealth, payment parity for telehealth and in-person services, and allowing clinicians to provide cross-state telehealth services. We found a significant decrease in the number of new buprenorphine treatment episodes in states that implemented telehealth parity laws after the declaration of the public health emergency. Telehealth’s potential for initiating buprenorphine treatment during COVID has been documented,^29,35,36,38,59,60^ and several studies have found that such state telehealth parity policies are associated with greater use of telehealth by patients and community mental health centers.^61–63^ We did find that buprenorphine treatment episode duration in 2020 was significantly longer than in 2019. One possibility is that longer treatment episodes may have decreased the availability of treatment “slots” for new patients if clinicians did not increase the total number of patients they were treating with buprenorphine. However, we are unaware of studies examining the effects of such policies on initiating buprenorphine treatment overall, initiating behavioral health treatment more generally, or the relationship between treatment duration of a clinician’s buprenorphine caseload and the number of buprenorphine treatment episodes they initiate.

We also found a significant increase in treatment duration for buprenorphine treatment episodes started in March 2020 before the declaration of the public health emergency, compared to the comparable period in 2019, consistent with several studies of the US Department of Veterans Affairs veteran population receiving buprenorphine.^36,60^ However, the only policy we examined associated with longer treatment duration was states implementing the psychologist interstate compact, which was associated with increased duration of treatment of approximately 7 days than treatment episodes in states that did not implement changes in the psychologist interstate compact. One possible explanation is that implementing such compacts may have increased access to non-pharmacologic mental health services in those states, thereby increasing access to counseling and psychotherapy, services which have been associated with longer buprenorphine treatment episodes.^44^ We are unable to observe such services in pharmacy claims, however, and additional research is needed to better understand the relationship between buprenorphine treatment duration and non-pharmacologic behavioral health services in the era of increased telehealth. We do note, however, that a variety of temporal chances may have also influenced adherence to buprenorphine treatment in the months after the declaration of the COVID public health emergency, such as changes in employment and associated changes in employer-provided health insurance, and decreased commuting time for those working from home and decreases in other out-of-home activities may have given individuals more time to keep appointments, particularly via telehealth.

Our findings must be considered in the context of the study’s limitations. We restricted our analyses to buprenorphine formulations indicated for OUD treatment, but we cannot identify off-label of those formulations (e.g., for pain). We are unable to observe telehealth use in the data, so are unable to directly assess the relationship of telehealth policies on telehealth use directly. The data also do not allow us to know the clinical setting in which prescriptions were written, the full duration of censored treatment episodes that are censored, the clinical status of patients to whom buprenorphine was dispensed, nor to what extent the characteristics on patients may have differed in the periods before and after the declaration of the public health emergency. We also do not know if our results generalize to prescriptions dispensed in pharmacies or hospital settings not captured in the IQVIA data or to prescriptions written but not dispensed. We also acknowledge other societal changes likely influenced healthcare-related behavior. Our cross-sectional analysis can only identify an association between states that implemented certain policies and the buprenorphine treatment we observe, and we have no information regarding how buprenorphine treatment evolved beyond the period we observe. Because of the limited time-horizon of our data (2019 to 2020), we were not able identify dynamic policy effects. Our policy effect estimates are identified assuming they are constant over time. If they are not, our estimates are almost certainly biased towards the zero. We have no information about variation in implementation or enforcement of these policies, the extent to which changes may have occurred in states that did not formally implement the policies, how long policies may have been in place prior to the declaration of the public health emergency, or how providers and payers interpreted and changed behavior in response to these policies. For example, insurers may have chosen to reimburse in-person and telehealth visits equally even in states not requiring parity. We also recognize that other state policies were implemented during this period as well as changes in federal policies; future research is needed examining how these changes may have influenced buprenorphine treatment patterns. Future studies are also needed to better understand how clinician’s perspectives regarding such policies influence their behavior, and how such perspectives and behaviors influence the effects of such policies.

## CONCLUSION

Despite these limitations, our findings contribute to the evolving literature regarding buprenorphine treatment during the first year of the COVID pandemic and the impact of state policies likely to facilitate telehealth. Buprenorphine and medication treatment for opioid use disorder continues to be a central pillar of the US response to the overdose crisis,^64^ with continued federal and state policymaker efforts to increase access to and use of buprenorphine.^65^ Consistent with existing literature, we find that buprenorphine treatment initiation was little changed subsequent to the declaration of the public health emergency, and duration of treatment was increased, in marked contrast to well-documented decreases in access and quality of care for individuals with mental health and substance use disorders more generally. However, given the increases in rates opioid misuse and overdose early in the pandemic, it remains unclear to what extent sustaining the amount of buprenorphine delivered was sufficient to address the need for medication treatment for OUD during that period.

We hypothesized that policy adaptations during the pandemic related to telehealth would increase initiation and retention in buprenorphine treatment; however, our findings did not support this hypothesis. Realizing the benefits of telehealth and other policy changes may require broader implementation and infrastructure support. Telehealth may provide substantial benefits for the individuals receiving it; however, benefits for the larger population receiving buprenorphine may be modest until additional factors are addressed such as access to technology or assistance locating a clinician who uses telehealth accepting new patients. As federal and state policymakers seek further flexibility in enhancing effective treatment for OUD, it is important to recognize that multiple system level investments may be needed to sustain access to care and increase the lifesaving potential of buprenorphine.

## Data Availability

The IQVIA Real World Data – Longitudinal Prescriptions used in this paper was analyzed under a Data Use Agreement with the authors and is not available from the authors. Individuals interested in use of the data should contact IQVIA.

## Appendix

See Table 4

**Table 4:** Baseline Buprenorphine Episodes and Treatment Duration by Presence or Absence of Each Policy in a State in 2019

	New buprenorphine episodes March 14 to December 31, 2019	2019 mean duration in buprenorphine treatment episodes March 1 to March 13, 2019
Telehealth parity before COVID		
Yes	475,227	138.1
No	105,704	139.9
Medicaid reimbursement for audio-only telehealth services before COVID		
Yes	529,666	138.4
No	51,265	138.1
Physician compact (IMLC) before COVID		
Yes	317,269	140.5
No	263,662	136.2
Psychologist compact (PSYPACT) before COVID		
Yes	487,601	139.3
No	93,330	135.0

## Declarations:

### Disclaimer:

The funders had no role in the design and conduct of the study; collection, management, analysis, and interpretation of the data; preparation, review, or approval of the manuscript; and decision to submit the manuscript for publication.
